# Growth Pattern Analysis of Murine Lung Neoplasms by Advanced Semi-Automated Quantification of Micro-CT Images

**DOI:** 10.1371/journal.pone.0083806

**Published:** 2013-12-23

**Authors:** Minxing Li, Artit Jirapatnakul, Alberto Biancardi, Mark L. Riccio, Robert S. Weiss, Anthony P. Reeves

**Affiliations:** 1 Department of Biomedical Sciences, Cornell University, Ithaca, New York, United States of America; 2 School of Electrical and Computer Engineering, Cornell University, Ithaca, New York, United States of America; 3 Institute for Biotechnology and Life Science Technologies, Cornell University, Ithaca, New York, United States of America; University of Navarra, Spain

## Abstract

Computed tomography (CT) is a non-invasive imaging modality used to monitor human lung cancers. Typically, tumor volumes are calculated using manual or semi-automated methods that require substantial user input, and an exponential growth model is used to predict tumor growth. However, these measurement methodologies are time-consuming and can lack consistency. In addition, the availability of datasets with sequential images of the same tumor that are needed to characterize *in vivo* growth patterns for human lung cancers is limited due to treatment interventions and radiation exposure associated with multiple scans. In this paper, we performed micro-CT imaging of mouse lung cancers induced by overexpression of ribonucleotide reductase, a key enzyme in nucleotide biosynthesis, and developed an advanced semi-automated algorithm for efficient and accurate tumor volume measurement. Tumor volumes determined by the algorithm were first validated by comparison with results from manual methods for volume determination as well as direct physical measurements. A longitudinal study was then performed to investigate *in vivo* murine lung tumor growth patterns. Individual mice were imaged at least three times, with at least three weeks between scans. The tumors analyzed exhibited an exponential growth pattern, with an average doubling time of 57.08 days. The accuracy of the algorithm in the longitudinal study was also confirmed by comparing its output with manual measurements. These results suggest an exponential growth model for lung neoplasms and establish a new advanced semi-automated algorithm to measure lung tumor volume in mice that can aid efforts to improve lung cancer diagnosis and the evaluation of therapeutic responses.

## Introduction

Lung cancer is the leading cause of cancer death among both men and women worldwide. The five-year survival rate for lung cancer is only 16%, as compared to 89% and 100% for breast and prostate cancers, respectively. If lung cancers are detected and treated in their earliest stages, the survival rate can be improved to 92% [1]. Not only is the detection of lung tumors critical, but measuring disease progression and treatment response also are important for improving patient care. Such clinical data can be collected using non-invasive imaging techniques, such as X-ray computed tomography (CT). CT images are generated based on X-ray attenuation by tissues, with the degree of attenuation proportional to the tissue density. CT instruments generate a series of 2D X-ray images, which can be reconstructed to produce a 3D image. Based on these images, clinicians can identify and measure potential lung neoplasms.

Tumor size and growth rate are key criteria for cancer staging and can be used to evaluate the effectiveness of therapies. Measurement of these parameters must be efficient and accurate in order for CT scans to be useful for clinical purposes. Manual measurements by clinicians have been used widely to assess tumor volumes in human patients. However, these methods are often time-consuming and labor intensive. Manual measurements are also subject to high inter- and intra-observer variability. Several studies have suggested that manual measurements of tumor size by radiologists are inconsistent [2], [3], [4] and should not be relied upon to provide ground truth. In response to these issues, semi-automated measurement methods have been developed to improve tumor measurement efficiency and reduce inconsistency among radiologists. However, the existing semi-automated methods typically require extensive manual intervention. For example, an algorithm described by Haines et al. [5] required the selection of the total chest space volume, excluding the heart, through a combination of manual segmentation and semi-automated contouring. In this case, tumor and vasculature tissue were not separated, and the combined volume of both was used as a relative measure of tumor burden. The measurement method developed by Fushiki et al. [6] also required manual segmentation of the chest volume. Cody et al. [7] used a method that did not require manual segmentation of the chest volume, but often required manual editing of the automatically determined contours. Namati et al. [8] described a semi-automated method that required only a single stroke across the cross-section of the tumor to initialize the algorithm, but also required manual editing of the automated segmentation. A recent study by Rodt et al. quantified tumor growth on longitudinal micro-CT scans. However, their measurement method required 20-40 manually specified seed points [9]. As a result, there has been an emerging need to develop a more advanced semi-automated method with minimal manual manipulation and no direct modification of the nodule segmentation boundaries.

The optimization of methods for tumor growth quantification requires not only time-efficient measurement tools but also their validation using extensive data sets from growing tumors. However, these data are not readily available for human patients because of the large number of repeated CT scans required and concerns over the associated X-ray dosage. Furthermore, human patients are subjected to clinical interventions that include surgical resection or therapies that impair growth. As a result, much of the research on evaluating the accuracy of measurement methods has relied on the use of repeat “coffee break” scans, in which patients are scanned twice over a short period of time [10], [11], [12], [13], or the use of repeat scans obtained during image-guided biopsy of pulmonary nodules [14]. These studies have reported measurement variability across repeat scans to be around 25%. It is unclear whether these data can be accurately extrapolated to define tumor growth properties.

One common approach to quantify tumor growth is to combine an empirical tumor volume measurement with a mathematical model for tumor growth rate. The two most commonly used growth models are the exponential growth model, in which the growth rate is proportional to the current value and continues indefinitely, and the Gompertzian growth model, which resembles the exponential growth model initially but reduces the growth rate as time progresses [15], [16], [17]. However, due to the reasons noted above, the *in vivo* growth of human lung cancers has not been extensively studied. In order to investigate the *in vivo* lung tumor growth rate, and to circumvent the limitations of human CT scans and current manual and semi-automated tumor volume measurement tools, we developed an advanced semi-automated method for the accurate determination of tumor volume for murine lung tumors imaged by micro-CT scanning. We hypothesized that the new semi-automated algorithm would efficiently and accurately measure lung tumor volume and produce measurement values that closely correlate with those from careful manual segmentation. In addition, because we examined early lung tumor growth when nutrients and space are not expected to be constraining for tumor growth, we hypothesized that an exponential growth pattern would be observed.

Micro-CT has previously been used to study lung disease in mouse models, including chronic inflammation, emphysema, and cancer [18], [19], [20], [21], [22], [23]. In this study, we utilized a mouse lung tumor model based on overexpression of the small subunit of the enzyme ribonucleotide reductase (RNR). RNR catalyzes the rate-limiting step in deoxyribonucleotide biosynthesis and plays an essential role in the maintenance of genome integrity. RNR-overexpressing mice develop lung adenomas and adenocarcinomas at approximately one year of age. These neoplasms histopathologically resemble human papillary adenocarcinomas, the most common form of human non-small cell lung cancer [24]. RNR-induced lung tumors frequently contain K-ras mutations, which also occur in approximately 30% of human lung adenocarcinomas [25], [26]. Unlikely currently used chemical-induced and activated K-ras-induced mouse lung tumor models [5], [6], [8], [27], RNR-induced lung tumors arise stochastically via a mutagenic mechanism caused by genome instability, providing a unique experimental system to investigate lung tumor growth rate *in vivo*.

In this study, initial imaging and volume measurements of mouse lung tumors were performed to develop and refine an advanced semi-automated algorithm adapted from tools for the measurement of pulmonary nodules in human chest CT scans [28]. This new semi-automated method had minimal requirement for manual manipulation and did not require any direct modification of the segmentation boundaries. The algorithm was first validated by comparing its output with post mortem physical measurements and manual image analyses. Lung tumor-bearing mice were then sequentially imaged by micro-CT for tumor volume measurement and growth rate determination. Tumor volumes measured by the advanced semi-automated method in sequential scans were compared with careful manual measurements, and close correlation between methods was observed, indicating the accuracy of the semi-automated algorithm. In addition, curve fitting of tumor volumes over time suggested exponential growth patterns for the analyzed lung neoplasms. Together, the results described here establish efficient and accurate tools for tumor volume measurement and provide insights into *in vivo* cancer growth properties that will be useful for future studies of animal models of lung cancer, including pre-clinical evaluation of candidate therapies.

## Materials and Methods

### Ethics statement

Mice used in this study were handled in strict accordance with federal and institutional guidelines. All procedures were approved by the Cornell University Institutional Animal Care and Use Committee (protocol number: 2008-0175). Every effort was made to minimize pain and distress to the animals during the studies. All mice were euthanized by carbon dioxide asphyxiation in accordance with American Veterinary Medical Association guidelines.

### Animals, necropsy and histology analyses

Rrm2 and p53R2 transgenic mice (Rrm2^Tg^ and p53R2^Tg^) were described previously [24]. All mice were maintained under identical housing conditions. After the final micro-CT scan, mice were euthanized by asphyxiation using carbon dioxide and necropsied. Lungs were photographed using a Canon digital camera, inflated and fixed with 10% neutral-buffered formalin, embedded in paraffin, sectioned, and stained with hematoxylin and eosin (H&E). H&E-stained sections were scanned using an Aperio ScanScope and physical measurements of lung neoplasms were made using Aperio ImageScope software. Lung neoplasms were classified based on guidelines endorsed by the Mouse Models of Human Cancers Consortium [29].

### Micro-CT imaging

#### Post-mortem imaging

Preliminary scans of mice following euthanasia were used for initial development and verification of the algorithm. All scans were acquired using a GE eXplore CT 120 micro-CT scanner, which has been evaluated previously [30]. Three-tumor bearing mice were scanned immediately after they were euthanized by carbon dioxide asphyxiation.

#### Live imaging

For live imaging, mice were first put in the induction chamber and anesthetized with a continuous flow of 4% isoflurane/oxygen mixture. When the animals stopped moving and their breathing slowed, they were moved to a Styrofoam platform in the cradle of the micro-CT scanner with their ventral side down. Mice were kept under anesthesia using a nose cone with a continuous flow of 1-3% isoflurane/oxygen mixture that was maintained during the duration of the micro-CT scan [31]. Respiratory gating of the mice was achieved using a BioVet physiological monitoring and triggering system (m2m Imaging Corporation), with a respiratory sensor positioned under the abdomen of the mouse. A small external force was applied by taping a piece of paper towel to the back of the mouse in order to facilitate activation of the pressure sensor used for respiratory gating. The trigger was set up at the quiescent/refractory period after each breath in order to minimize motion artifacts.

All scans of the thoracic region were acquired using a GE eXplore CT 120 micro-CT scanner with a current of 50 mA, voltage of 100 kVp, exposure time of 20 ms, and acquisition resolution of 50 µm. Each scan consisted of 720 projections in a single full rotation of the gantry. Two frames were acquired at each position of the gantry and averaged prior to being transferred to the workstation for reconstruction (Table S1). Micro-CT images were reconstructed at 50×50×50 µm^3^ voxel dimensions. The final image volume varied according to the selected region of interest but typically ranged from 20×20×25 mm^3^ to 40×27.5×50 mm^3^. Half of the total live scans included calibration phantoms with materials of known densities simulating air, water, and bone. The densities of the voxels within the phantom were sampled and the mean and standard deviation of the distribution were computed. Scans were converted from the manufacturer's proprietary format to DICOM and then imported into research software. Scan data are available for downloading at: http://www.via.cornell.edu/microdb.html.

The four lung tumor-bearing mice in the study were scanned a minimum of three times each, with at least three weeks between scans. The resulting images were examined to identify potential nodules. For each mouse, at least one tumor was visible in every scan over the course of the study and was tracked sequentially for growth rate analysis. Only tumors that were visible on three or more sequential scans were considered for inclusion in the longitudinal study to allow for fitting of a growth curve. The volume of each nodule was computed manually and by the semi-automated algorithm described in the following sections at each time point and recorded for growth analysis. Two observers independently verified the segmentation of the tumors by the algorithm to make sure the measurements from the semi-automated algorithm were reliable. Mice were euthanized following the last live scan, and necropsy and histological analyses were performed for further characterization of the tumors identified in micro-CT scans.

### Semi-automated volumetric analysis

#### Algorithm overview

**Figure 1:**
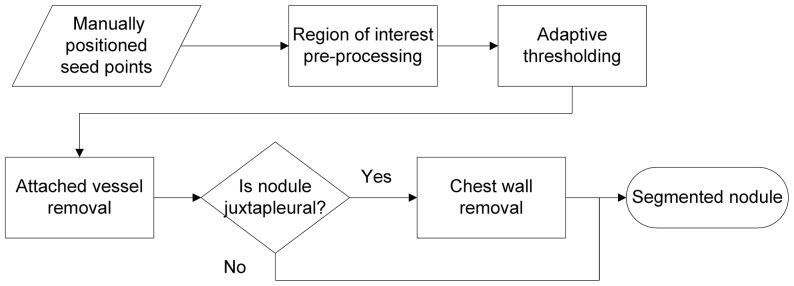
Flowchart showing the major steps of the semi-automated segmentation algorithm.

The volume of the murine lung tumors was computed from the segmentation result produced by a semi-automated algorithm. The algorithm was adapted from previous work on semi-automated segmentation of lung tumors in whole lung CT scans of humans [28]. An overview of the algorithm, which consists of several steps, is presented in Figure 1. The user initiates the algorithm by drawing a line across the nodule indicating the nodule's extent. The algorithm uses the provided information to extract a region of interest to analyze. After pre-processing the image, an adaptive threshold is applied, followed by filtering to remove attached pulmonary vessels. The lung regions and their volumes are computed to aid in determining if the nodule is juxtapleural; if so, an additional step is performed to separate the nodule from the chest wall. The final result is a binary image containing only those voxels belonging to the nodule.

#### Algorithm modifications to enable micro-CT data analysis

The algorithm used to analyze murine micro-CT scans was adapted from tools for the measurement of pulmonary nodules in human chest CT scans [28]. To adapt the algorithm to analyze murine micro-CT scans, several changes were required. First, in micro-CT scans, the size of the extracted region of interest of the scan was clipped to a 5×5×5 mm^3^ cubic region of interest (ROI) around the location of the tumor that was manually provided to the algorithm.

**Figure 2:**
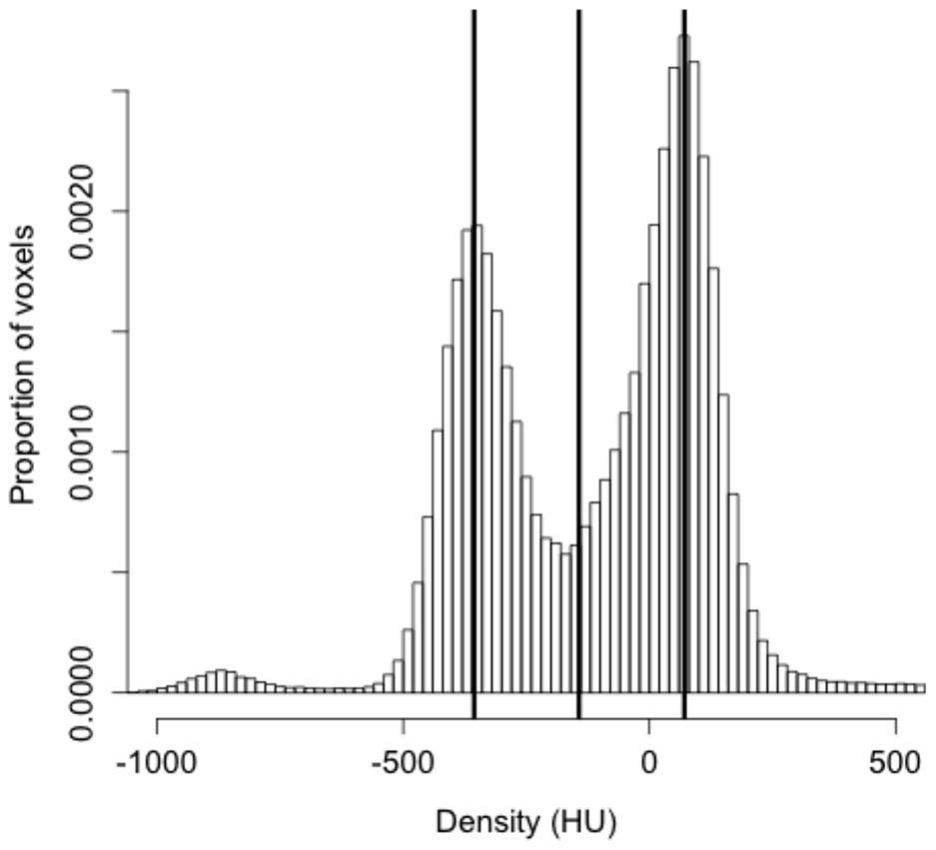
Histogram showing voxel densities of the lung parenchyma and soft tissue in a region of interest. Voxel densities of a region of lung from an RNR transgenic mouse including a nodule were sampled and used to create the histogram. Bins were set at 10-automated algorithm. The data shown were from a scan taken at 50 µm with 720 projections.

Second, the method for determining the threshold used to segment the soft tissue from the lung parenchyma was modified to account for the variation in calibration in the micro-CT scans. After the micro-CT scan is clipped, a threshold is applied to the ROI to separate the soft tissue from the lung parenchyma. While the use of an adaptive threshold does not provide much benefit for segmenting nodules from human CT scans compared to a fixed threshold [32], the murine micro-CT scans exhibited significant variation in the density of the lung parenchyma from one scan to another. To compensate for this variation, an adaptive threshold was selected for each scan. A histogram of the ROI, prior to resampling, was generated. A threshold was established based on the assumption that the histogram would be bi-modal, with one peak corresponding to the lung parenchyma and another to the soft tissue. The threshold was automatically selected to be the midpoint of the two peaks, as shown by the plot in Figure 2. After the adaptive threshold was determined, the threshold was applied to the resampled ROI image to generate a binary image, with all soft tissue structures labeled.

The remaining steps of the segmentation algorithm were the same as the one used for human chest CT scans [28]. After the completion of the algorithm, the completed segmentations were manually reviewed. If the segmentation is not acceptable, several parameters can be adjusted from the automatically determined values: the size of the processing filter used to remove vessels; the size of the region of interest, if there is an excessive or insufficient amount of the chest wall; and whether or not the tumor is juxtapleural. These parameters are only adjusted relative to the algorithm-derived values and typically only require a small adjustment. The size of the vessel processing filter controls the extent of vessel-like structures that are included in the segmentation of the tumor. The size of the ROI is set to be large enough to totally encompass most nodules and provide a sufficient portion of the chest wall to allow for the correct function of the surface fitting algorithm, but not any larger than necessary to limit the computation time and avoid including other non-tumor structures. In some cases, the ROI had to be increased by 1.0 mm for nodules that were larger than the ROI.

### Manual tumor measurements

The tumor volumes measured by the semi-automated algorithm were compared to those generated by manual measurement procedures. The approximate volume of all tumors in the study was measured manually using the following method. In every fifth slice through the tumor, an observer performed a bi-dimensional measurement consisting of a line drawn along the greatest extent of the tumor and a perpendicular line that spanned the greatest extent of the tumor in the other dimension. The average of these two measurements was used to establish the diameter of a cylinder with a height of 5 slices (0.25 mm). In this manner, manual measurements were made across the full thickness of the tumor, and the resulting volumes were summed to obtain an approximation of the nodule volume. In order to validate this manual approximation of nodule volumes, we also measured the volumes of a subset of tumors by complete manual 3D segmentation in which the tumor boundaries were outlined manually in all slices.

### Growth analysis

In this study, each mouse was scanned several times. Tumors were identified and their volumes were measured for each scan. Tumor growth can be determined from the tumor volume and time interval between scans. Exponential growth can be modeled by the following equation: 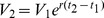
where V_1_ is the volume at time t_1_, V_2_ is the volume at time t_2_, and r is the growth rate.

A nonlinear regression curve fitting was performed using GraphPad Prism. The exponential growth equation analysis gave the tumor growth rate *r* for all the tumors studied.

The tumor volume doubling time (VDT, defined as ln2/r), as well as the growth index (GI), was calculated for tumors observed in the scans. GI, the percent tumor growth per month, was defined as follows:
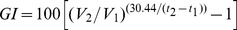



The value 30.44 is the average number of days per months. Rearranging this equation gives a GI value that can be calculated using the slope r of the best-fit exponential curve:



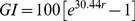
(3)


In contrast to VDT where a lower value indicates faster growth, a higher GI represents faster growth. VDT can be converted to GI by the following equation:
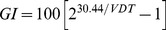



The manual and semi-automated methods for calculating tumor volumes were compared by plotting ln (volume) versus time and obtaining the best-fit lines. The slopes of these lines were compared by Student's t test. The R-squared values were used to evaluate the goodness of fit with a 95% confidence interval.

## Results

**Figure 3:**
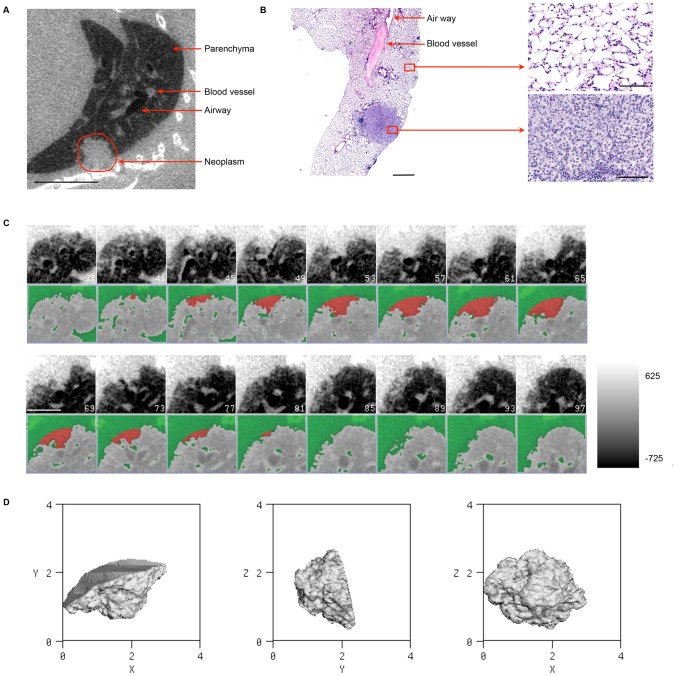
Micro-CT and histological analyses of an RNR transgenic mouse lung tumor. (A) Micro-CT image of lung (sagittal view) from an RNR transgenic mouse with a tumor (red circle generated manually). Image was derived from a scan taken at 50 µm with 220 projections. The scale bar represents 5.0 mm. (B) H&E stained lung tissue from the same mouse. The scale bar represents 1000 µm. Normal and tumor tissues are also shown at a higher magnification. The scale bar represents 40 µm. The tumor diameter was measured to be 1.94 mm by histological analysis. (C) Black and white panels: Several slices (every 4th slice shown) through a small region of interest including the tumor in (A). The scale bar represents 2.5 mm. Color panels: The same tumor separated by the semi-automated segmentation algorithm from other soft tissue structures such as blood vessels and the chest wall. The result is shown with the tumor in red and other soft tissue structures in green. The color bar range is −725 to 625 HU. (D) A 3D visualization of the segmented tumor in (C) showing axial, sagittal, and dorsal views. The volume equivalent diameter of the tumor was calculated by the semi-automated algorithm to be 2.03 mm.

In this study, we used micro-CT to monitor the progression of murine lung tumors *in vivo*. Prior to imaging live mice, post-mortem imaging of three lung tumor-bearing mice was performed in order to optimize and validate the measurement algorithm. Figure 3A shows a micro-CT image of the lungs from a euthanized mouse scanned with 360 projections, reconstructed at 50×50×50 µm^3^ resolution. The neoplasm apparent in the micro-CT scan was also identified on the histological slide of the corresponding lung lobe (Figure 3B). The neoplasm is an adenoma, with a clear border between the solid tumor mass and surrounding lung parenchyma. Higher magnification images of the histological section clearly showed that the tumor region is much denser than the surrounding lung parenchyma, hence the different densities of these tissues observed in micro-CT. We then compared tumor size as determined by physical, image-based manual, and semi-automated computational approaches, using maximum tumor diameter as the readout that could be most readily assessed using all three measurement methods (Table S2). The diameter of this tumor was determined to be 1.94 mm by physical measurement of a histological section. Manual measurement of micro-CT scans was used to determine the volume of the same tumor, and the diameter was calculated to be 2.06 mm. Next, the semi-automated algorithm was used to measure the size of the same tumor. The ROI including the tumor is illustrated in Figure 3C, with every fourth image from the tumor segmentation shown. A 3D visualization of this segmented tumor was then generated with axial, sagittal and dorsal views shown in Figure 3D. Consistent with the histological and manual measurements of the tumor, the diameter computed by the algorithm was 2.03 mm. Physical, manual and computed measurements for three additional tumors from two tumor-bearing mice were compared and also found to be highly similar, with the difference in tumor diameter between the manual and semi-automated measurement methods ranging from 1.47% to 4.49% (Table S2). Together, these results provided an initial validation of our computational methods for semi-automated measurement of mouse lung tumors from micro-CT images.

Live micro-CT scanning was then performed on four lung tumor-bearing mice. All live scans were acquired using the following parameters: 100 kVp, 50 mA, 20 ms, 720 projections, 2 frame averaging and acquisition and reconstruction at 50×50×50 μm^3^ resolution, with the exception of two scans (Table S1). 12 tumors in total were detected in the four mice, and ten tumors were successfully segmented by the semi-automated algorithm (Table S3). Each mouse was euthanized following the final scan, and necropsy and histological analyses were performed to confirm tumors that were detected by micro-CT scans. Of the ten successfully segmented tumors, six were detectable at three or more time points, permitting their use for growth analysis. To validate the accuracy of the algorithm in live studies, we first compared the volumes of the four nodules not used for growth analysis as measured by the algorithm with the volumes measured for the same tumors manually (Table S4). Consistent with the findings from post-mortem scans indicating that the semi-automated algorithm was accurate, no significant difference was observed between volume measurements made by the algorithm or manually for the tumors from these live imaging studies (Table S4, paired Student's t-test, P = 0.57).

**Figure 4:**
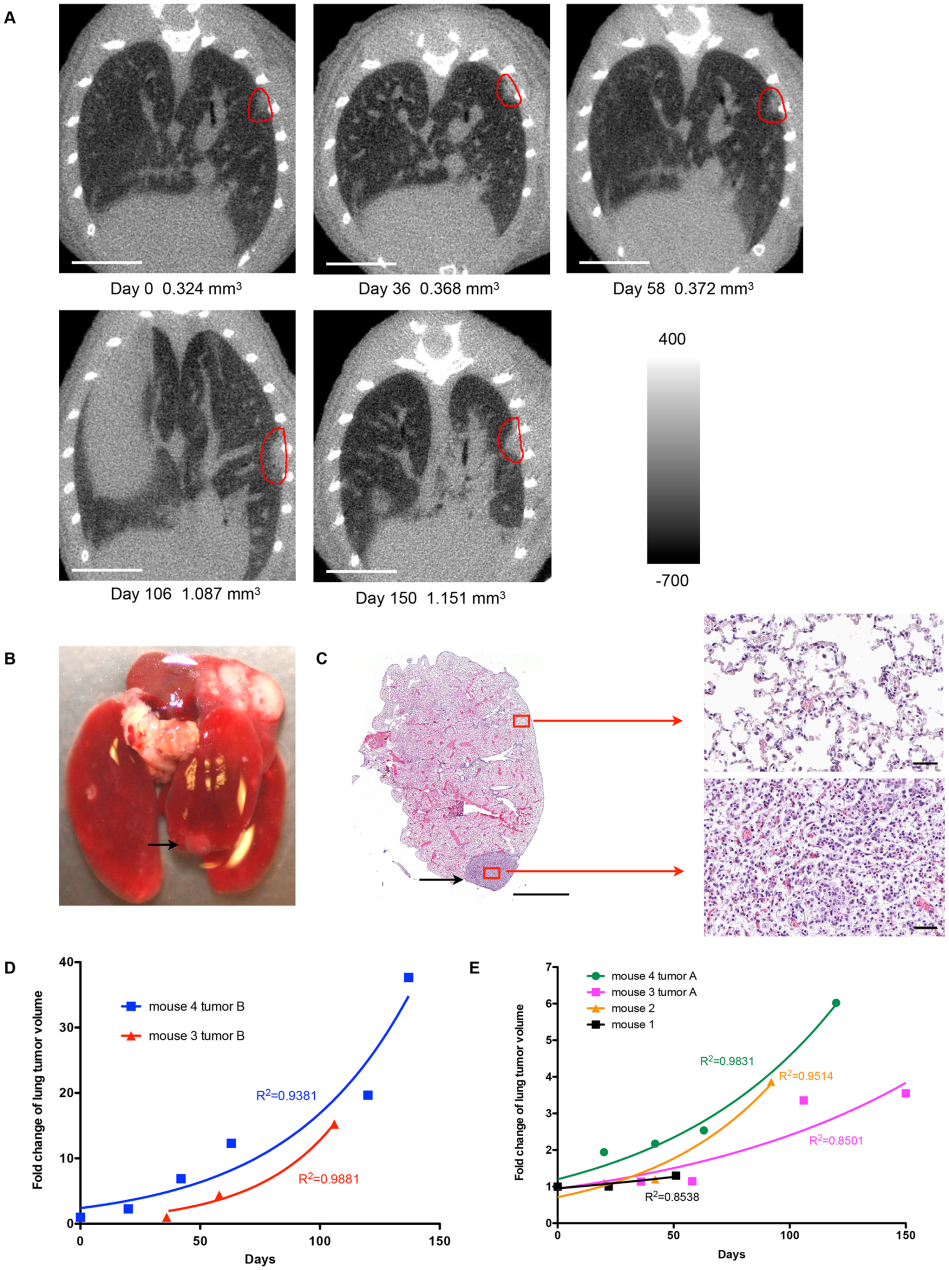
Sequential micro-CT scans over time to measure lung tumor growth rate in four RNR transgenic mice. (A) Images of sequential micro-CT scans of an RNR transgenic mouse (mouse #3 tumor A; red circle generated manually). Images were acquired at 50 µm with 720 projections. The scale bars represent 5.0 mm. The color bar range is −700 to 400 HU. (B) Gross image of the lungs at necropsy showing the tumor (black arrow) after the last scan. (C) H&E stained section from lungs shown in (B). The scale bar represents 1000 µm. Normal and tumor tissues are also shown at a higher magnification. The scale bar represents 40 µm. (D, E) Growth curves of lung tumors from four RNR transgenic mice. Fold change in lung tumor volume was plotted against time from the first micro-CT scan. A best-fit exponential curve was used to model the growth of each tumor. Note that Mouse 1 showed very slow growth, which could be due to inconsistency in tumor volume measurement because different scan parameters were used for mouse 1 time point 3 and this was the first live mouse scanned, when the micro-CT instrument was not calibrated for each scan.

**Figure 5:**
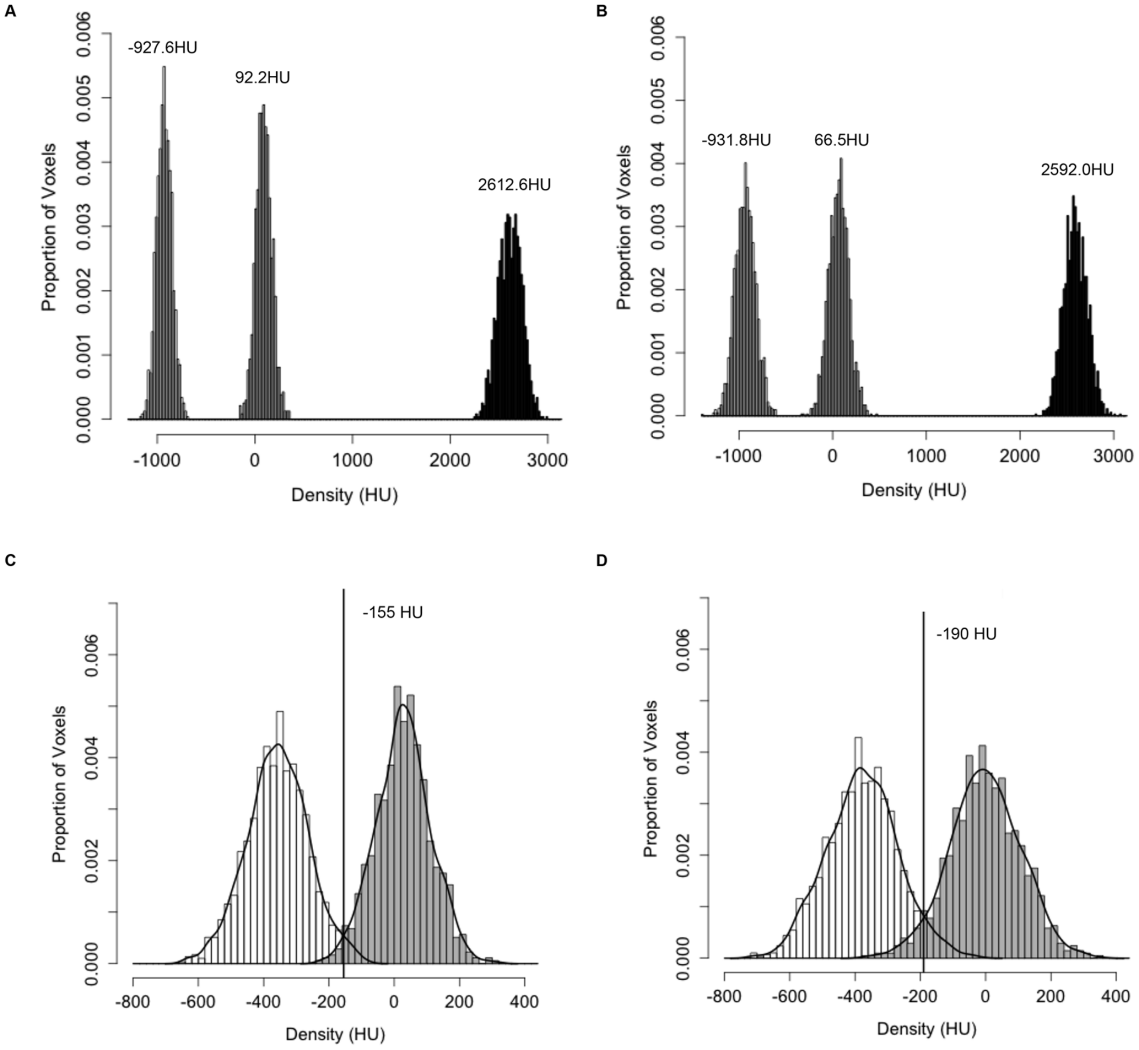
Phantoms and tissues show variation in densities across different scans. (A) Distribution of densities of three phantoms, air, water, and bone (left to right peaks), with mean densities −927.6 HU, 92.2 HU, and 2612.6 HU, respectively, in one scan. (B) Distribution of densities of the same phantoms as in (A) in a repeated scan six weeks later with mean densities −931.8 HU, 66.5 HU, and 2592.0 HU, respectively. (C) Distribution of densities in the lung parenchyma (white) and soft tissue (gray) from one mouse in one scan with an adaptive threshold at −155 HU. (D) Distribution of densities of the same tissues as in (C) of the same mouse in a repeated scan six weeks later with an adaptive threshold at −190 HU. All scans were acquired at 50 µm with 720 projections. Variations in the density distribution of the phantoms and tissues were observed in repeated scans.

Next, the volumes of the six nodules used in growth analysis were computed at each time point and recorded for growth pattern analysis (Figure 4A to C, Figure S1, and Table S5). The fold change in tumor volume over time for each nodule is illustrated in Figure 4D and 4E. The initial volume for the tumors in Figure 4D was approximately 0.050 mm^3^ while the initial volume for the tumors in Figure 4E ranged from 0.190 mm^3^ to 0.898 mm^3^ (mean 0.3665 mm^3^, median 0.257 mm^3^). The growth index (GI) and volume doubling time (VDT) for each tumor is provided in Table 1. The growth curves of the tumors in Figure 4 were suggestive of an exponential growth pattern, with deviation from a perfect exponential model. Variation from the micro-CT scanner due to alterations in calibration from scan to scan could contribute to such a deviation. This possibility was assessed using data for phantoms of known density that were scanned together with the mice. Histograms for the distribution of densities for air, water, and bone phantoms were plotted for two scans of the same mouse (Figure 5A, B), with mean and standard deviation values reported in Table S6. While the mean density of the air phantom between the two scans only varied by 4.2 Hounsfield Units (HU), the means for the water and bone phantoms differed by 25.7 HU and 20.6 HU, respectively. The noise of the scans, which was quantified by the standard deviation of the distribution of pixel values in the phantoms, also differed from scan to scan. The greatest difference in standard deviation between the two scans occurred with the air phantom, where there was a 30.9 HU difference. The difference in standard deviation between the two scans decreased as the mean density increased, with the difference for water and bone being 24.7 HU and 3.4 HU respectively. In addition to phantoms, we also measured the voxel densities of the lung parenchyma and soft tissue from different scans to see whether they were consistent. Figure 5 shows the distribution of voxel densities for the lung parenchyma and soft tissue from two different scans of the same mouse (mouse 4; Figure 5C and 5D). The value that best separated the lung parenchyma from soft tissue varied by 35 HU from one scan to the other. These data identify scan-to-scan variability in calibration and tissue density measures.

**Table 1:** Tumor volume doubling time and growth index of tumors detected in RNR transgenic mice by micro-CT.

Mouse (Tumor)	Age at first scan (month)	Initial tumor volume (mm^3^)	Growth curve slope	Tumor volume doubling time ( = ln2/slope) (days)	Growth index [ = 100(e^30.44*slope^-1)] (% increase per month)
1	11	0.685	0.0057	121.6	18.95
2	12	0.898	0.0182	38.08	74
3(A)	12	0.324	0.0096	72.2	33.94
(B)		0.052	0.0297	23.34	147.0
4(A)	11	0.190	0.0134	51.73	50.36
(B)		0.050	0.0195	35.55	81.04

NOTE: mice were first scanned at the indicated age and then subjected to a series of sequential scans to monitor tumor growth. The slope of the growth curve was converted to tumor doubling time and growth index to indicate the rate of tumor growth.

Next, we investigated whether differences in mouse positioning or disease progression could contribute to measurement variation between scans, possibly by affecting nodule morphology. This possibility was assessed by comparing total lung volume across all scans for each mouse. The difference between the minimum and maximum lung volume measurement was 43.7% (mean volume 524.0 mm^3^, standard deviation 82.3 mm^3^) for mouse 2, 60.8% (mean volume 484.3 mm^3^, standard deviation 79.9 mm^3^) for mouse 3, and 28.2% (mean volume 576.6 mm^3^, standard deviation 57.7 mm^3^) for mouse 4. These findings indicate that there were substantial changes in total lung volume for individual mice between imaging sessions.

**Figure 6:**
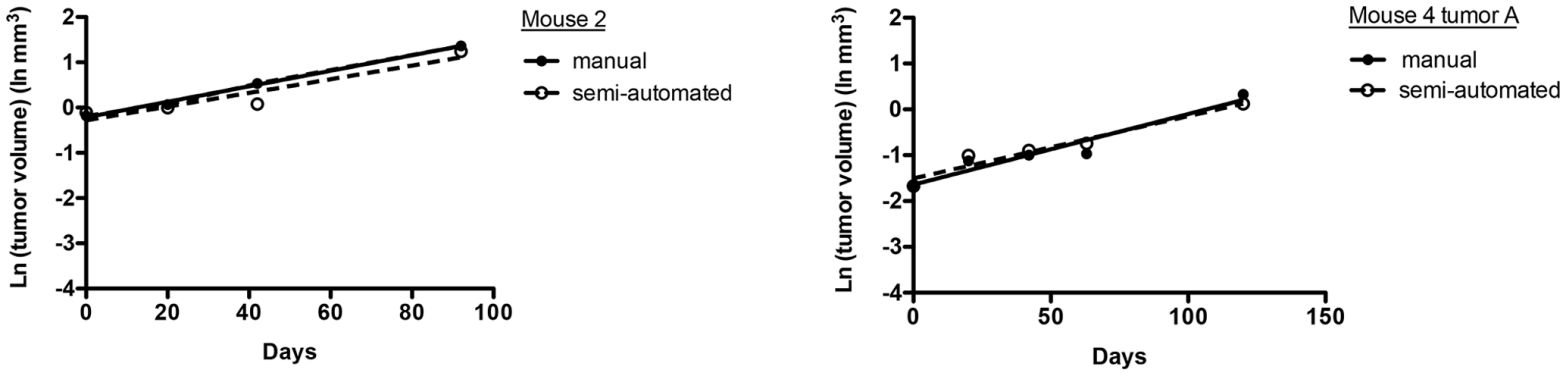
Comparison of lung tumor growth measured manually by an observer and by the semi-automated algorithm. Best linear fit growth curves were plotted for tumors from mouse 2 (left) and mouse 4 tumor A (right) based on measurements by a manual approximation method and by the semi-automated algorithm. The slopes of the best-fit lines for the manual and semi-automated measurements were compared by Student's t-test, and no significant differences were observed between the two slopes (P = 0.62 for mouse 2 and P = 0.57 for mouse 4 tumor A).

In addition, inaccuracy of tumor volumes measured by the semi-automated algorithm could also contribute to deviations in the exponential model. To test whether the algorithm accurately measured tumor volume changes over time, we compared the change in tumor volume over time as measured manually and as computed by the algorithm, for each of the six nodules used in the tumor growth analyses. Two different manual measurements were used, based on either complete manual segmentation of each tumor or a simplified approximation method for manual measurement as described in the Materials and Methods section. No significant differences were observed for values from any of these three measurement methods (Table S5, one-way ANOVA, P = 0.06). In addition, because the tumors showed exponential growth, the tumor volumes from both measurements were converted to a log scale and the resulting best-fit lines were compared (Figure 6 and Figure S2). Statistical comparison of the slopes of the best-fit lines revealed no significant differences between the values from semi-automated and manual measurements (see legend of Figure S2 for P-values), confirming that the semi-automated algorithm accurately measures tumor volumes *in vivo*.

## Discussion

The development of micro-CT has provided an opportunity to monitor disease progression and therapeutic responses over time in mouse models of human diseases. To further explore this opportunity and improve the measurement tools, we developed a semi-automated method to measure tumor volume in micro-CT scans from a mouse lung cancer model. Several studies have examined *in vivo* tumor growth in mouse models imaged with micro-CT using semi-automated measurement methods. These typically required manual editing of the segmentation boundary [7], [8] or a large number of manually specified seed points [9]. In contrast to existing semi-automated methods, the semi-automated method in this study only requires a single stroke across the nodule on the central slice, and optionally allows for manual adjustment of three parameters: the size of the processing filter used to remove vessels; the size of the region of interest, if there is an excessive or insufficient amount of the chest wall; and whether or not the tumor is juxtapleural. This advanced semi-automated method required minimal manual intervention, and manual boundary modification is not allowed, making it more efficient and less labor intensive. Another recent report described an automated method for lung nodule segmentation from micro-CT images that, like the approach reported here, also enables accurate tumor volume measurement in longitudinal studies [33].

**Figure 7:**
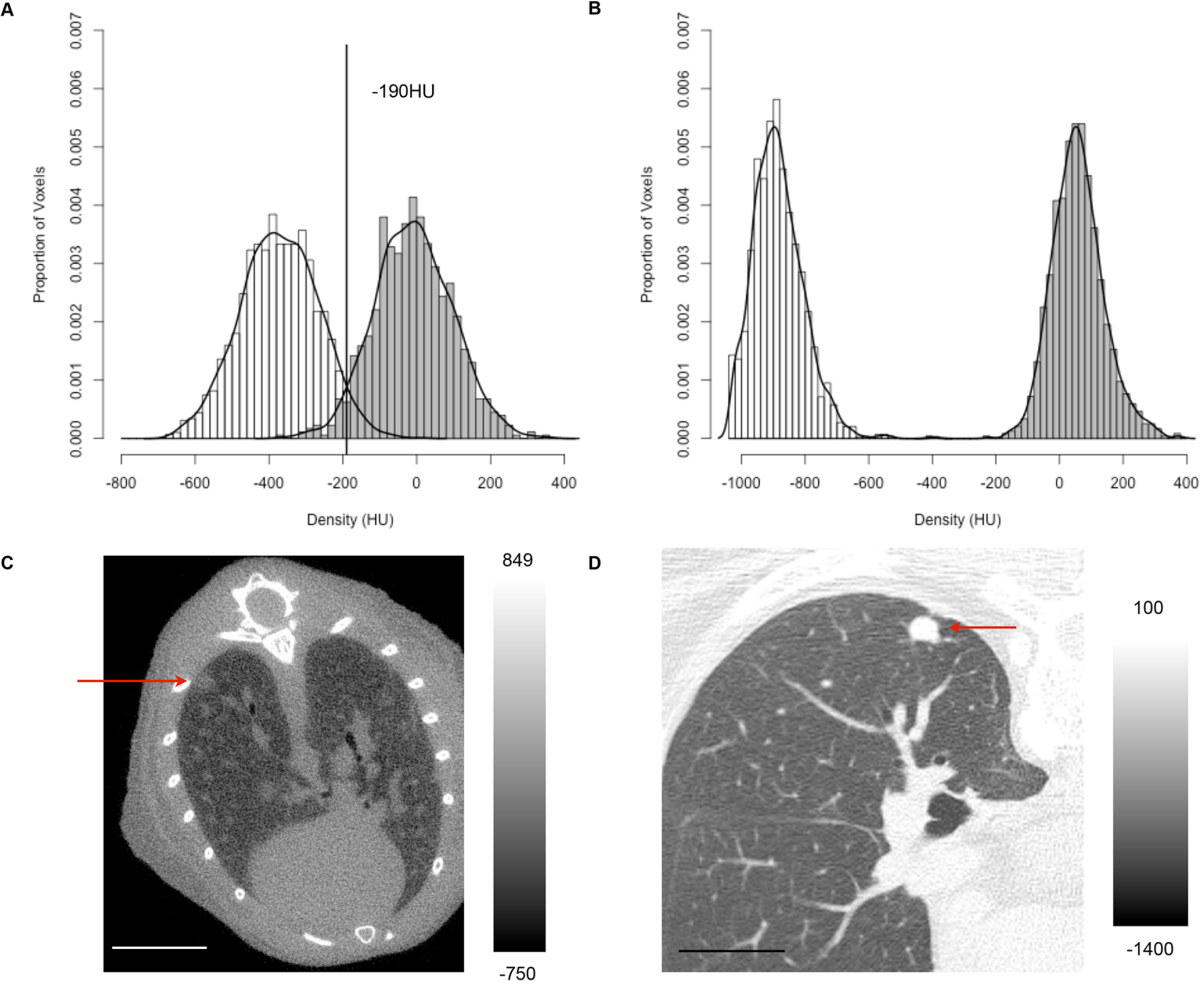
Comparison of soft tissue and lung parenchyma densities in a micro-CT scan and a human whole lung CT scan. Distribution of densities in the lung parenchyma (white) and soft tissue (gray) in (A) a mouse micro-CT scan with adaptive threshold of −190 HU and (B) a human whole lung CT scan with no need for adaptive threshold. The mouse micro-CT scan was obtained at 50 µm with 720 projections. The human whole lung CT scan was from the Weill Cornell Medical College Lung CT database. It was obtained using a GE LightSpeed Ultra scanner at 120 kVp and 80 mA, with 0.7×0.7×1.25 mm^3^ resolution. The peaks in (A) were not as sharp as those in (B), indicating that the mouse micro-CT scans were noisier than human CT scans. Magnified regions of the lung from (C) a micro-CT scan (yellow circle indicates tumor) and (D) a whole-lung CT scan (red arrow points to tumor) are shown to visualize the difference in scan quality. No scaling was done to the images and each image was windowed for viewing. The scale bars represent 5.0 mm (mouse micro-CT image) or 70.3 mm (human CT image). The color bar range is −750 to 849 HU (mouse micro-CT image) or −1400 to 100 HU (human CT image).

Micro-CT scans present an additional challenge for segmentation algorithms compared to clinical human CT scans. Although we used a set of scan parameters that provided the least amount of noise given our radiation dosage limitations, the noise level in these micro-CT scans was still higher than what is typically obtained using low-dose whole lung human CT scans for which the algorithm was originally designed. This is due in part to the scale at which micro-CT operates – the image noise is inversely proportional to the spacing between the voxels, if X-ray exposure is held constant [34]. The micro-CT scans in this study were acquired with a voxel size of 0.05×0.05×0.05 mm^3^ compared to typical human CT scans of 0.6×0.6×1.0 mm^3^. Figure 7 shows a single snapshot of a typical low-dose human CT scan obtained from the Public Lung Database to Address Drug Response as well as a micro-CT scan obtained in this study. Visually, the micro-CT scan has a noisier appearance, as evidenced by the texture pattern of the lung parenchyma compared to that of the human CT scan (Figure 7C and 7D). The increase in noise is reflected in the histogram shown in Figure 7A, in which the micro-CT scan has a much wider distribution of densities than the human CT scan shown in Figure 7B. The mean and standard deviation of the distributions are given in Table S7. Nevertheless, the semi-automated algorithm developed in this study is able to cope with the noise level found in micro-CT scans, as illustrated by the successful segmentation of tumors in Figure 3D.

The semi-automated algorithm used in this paper successfully segmented 10 out of 12 tumors detected in live scans of four mice. One limitation the semi-automated algorithm has is that it cannot properly segment tumors that have complicated morphology. One assumption made during the development of the algorithm was that the tumor would only touch the outer chest wall. As a result, two tumors that were located in the lower lung near the diaphragm and touched both the chest wall and the diaphragm could not be successfully segmented (Figure S3).

The growth curves of the tumors followed an exponential pattern, which is consistent with modeling of tumor growth patterns for human lung cancers [35], [36]. It is also consistent with a recent CT study showing exponential growth in early stage pancreatic cancers [37]. Therefore, our *in vivo* murine lung tumor growth study is consistent with the exponential growth model of early lung tumors, for which nutrients, gases and physical space are not limiting, and supports its use in tumor measurement algorithms to monitor disease progression and therapeutic responses. At larger tumor sizes, cell division no longer proceeds in an unconstrained fashion and non-exponential growth patterns have been reported [16], [17], [38]. It also should be noted that the growth curves obtained in this study showed deviations from an ideal exponential model, although it remains unknown whether this accurately reflects the actual growth behavior of the tumors. We found that alterations in scanner calibration and changes in mouse lung volume are factors that could contribute to measurement variability.

One characteristic of CT is that the scale is calibrated. Air has a density of −1000 HU and water has a density of 0 HU. Thus, materials of the same density should have a consistent density in CT scans, both across time and instruments. This allows for the use of a fixed threshold to separate different tissue types, in this case for the lung parenchyma and soft tissue. However, in this study, we observed inconsistencies in calibration and noise characteristics from scan to scan that could contribute to measurement variation and deviation from a perfect exponential growth curve. The semi-automated algorithm in this study used an adaptive threshold estimated from the local ROI instead of a fixed threshold to address this variation in calibration. These findings additionally suggest that the micro-CT scanner should be calibrated prior to each scan using density phantoms that span the entire range of densities to be observed in the scan.

We also observed changes in lung volume between live scans for individual mice. The lung volume of the same mouse varied up to 60% from one scan to another as measured by the algorithm. It is currently not possible to determine how changes in total lung volume might affect tumor volume measurements; we cannot rule out the possibility that differences in lung volume could affect tumor volume measurements because of the associated variation in distending forces from the surrounding parenchyma. Slight differences in mouse positioning between scans could affect the shape and inflation of the lung, which in turn could contribute to observed differences in lung volume. In addition, as lung disease progressed, the increased tumor burden resulted in reduced respiratory capacity, which might be another factor contributing to changes in observed lung volume. Furthermore, in order for the mouse to activate the pressure sensor used for respiratory gating during live scans, a small external force was applied on the back of the mouse, which could lead to slight compression of the lungs. An alternative method for respiratory gating is to use a ventilator to control the respiratory cycle of the mouse [39]. This would eliminate the need for a pressure sensor and provide better control of the degree and rate of inspiration.

Manual tumor volume measurements were made to provide validation of the semi-automated method. Although uni- and bi-dimensional measurements on a single slice are typically performed in clinical practice [40], such as the method specified by Response Evaluation Criteria In Solid Tumors (RECIST) [41], we performed bi-dimensional measurements on every 5^th^ slice throughout the tumor to improve the accuracy of the manual measurements and used a cylindrical approximation to estimate the tumor volume without requiring a full manual segmentation of the tumor. Since this method is not typically used, we validated this manual measurement against a full manual segmentation of a subset of the tumors. As shown in Figure S2, there was good agreement between complete manual segmentation and the bi-dimensional manual approximation.

The neoplasms analyzed in this study were all adenomas. The GI values for these tumors ranged from 18.94 to 146.9% per month (mean 67.55%, median 62.18%), which is much greater than the GI of 5.4% per month reported for human lung tumors [42], indicating that the murine tumors grow faster. This likely is due at least in part to the fact that, relative to larger human tumors, the tumors in mice were detected at an early stage when they experience preferential growth conditions, including greater nutrient access, oxygenation and less spatial constraint. Similarly, tumors with smaller initial volumes in this study tended to grow faster, although the limited sample number in our study of lung neoplasms precludes definitive conclusions on this point. Haeno *et al.* [37] previously reported that primary pancreatic tumors that were larger at diagnosis grew slower. Tumor VDT for the tumors analyzed in this study ranged from 23.34 to 121.6 days (Table 1), with an average of 57.08 days. These values are similar to published VDT values for mouse lung adenocarcinoma. Oliver *et al.* [43] reported an average mouse lung tumor VDT of 35 days (with high variation). Haines *et al.* [5] and Fushiki *et al.* [6] showed an average VDT for murine lung tumors of around 42 days. These published studies all used the K-ras^LSL-G12D^ mouse model, in which expression of the activated K-ras oncogene is induced following Adeno-Cre viral infection [44]. In comparison, lung tumors arise in the mouse model used in the present study through a stochastic process that also is associated with mutations in the K-ras oncogene [24]; differences between the mouse models used may contribute to the modest variation in average tumor VDT observed. A recent study in human lung cancer patients revealed an average tumor VDT of 136 days for 111 lung cancer cases [45]. 110 of the 111 cases had lung tumors diagnosed at screening rather than by symptoms, suggesting that the average VDT of 136 days reflects tumor growth at an early stage and provides a basis for comparison with the average VDT of 57.08 days in our mouse model. Aside from cancer cell-intrinsic factors, previous research has identified the tumor microenvironment provided by the host, including capillary density and metabolic activity in surrounding tissues, as a key, species-specific determinant of tumor growth rate, with mouse tissue typically supporting more rapid tumor proliferation [38], [46].

The semi-automated pulmonary nodule segmentation algorithm for measuring murine tumors imaged by micro-CT reported here was capable of accurate measurements of tumor volumes and was used to monitor disease progression over time. Tumor volume measurements from micro-CT have the potential to be used as an imaging biomarker in preclinical studies. With future improvements to handle nodules with more difficult morphology, this automated algorithm holds promise for use in monitoring disease progression following treatment with candidate drugs and evaluation of therapeutic responses.

## Supporting Information

Figure S1 **Analysis of lung tumor growth in RNR transgenic mice by sequential micro-CT scanning.** Micro-CT imaging was performed on RNR transgenic mice, and representative micro-CT images from each imaging session are shown for (A) mouse #1; (B) mouse #2; (C) mouse #3 tumor B; (D) mouse #4 tumor A; (E) mouse #4 tumor B. All images were acquired at 50 µm with 720 projections. The time point of the scan and the calculated tumor volume are indicated. Manually generated red outlines highlight the tumor analyzed in each image. Also shown are H&E stained sections that were generated for each sample following the final imaging session. Scale bars represent 5.0 mm (CT images) or 1000 µm (H&E image). The color bar range is -800 to 500 HU.
(TIF)

Figure S2 **Comparison of lung tumor volume values determined manually by an observer and by the semi-automated algorithm.** Four tumor-bearing mice were subjected to sequential micro-CT imaging, and tumor volume measurements of six tumors were determined manually by an observer and by the semi-automated algorithm. (A) mouse #1, (B) mouse #2, (C) mouse #3 tumor A, (D) mouse #3 tumor B, (E) mouse #4 tumor A, and (F) mouse #4 tumor B. The volumes of all six tumors were measured over time by a manual approximation method as described in Materials and Methods. The slopes of the plotted lines for the manual approximation and semi-automated measurements were compared by Student's t-test, and no significant differences between the two slopes were observed (mouse 1: P = 0.40; mouse 2: P = 0.62; mouse 3 tumor A: P = 0.99; mouse 3 tumor B: P = 0.69; mouse 4 tumor A: P = 0.57; mouse 4 tumor B: P = 0.55). In addition, the volumes of three tumors over time were further measured by manual complete 3D segmentation (mouse 2, mouse 3 tumor A and mouse 4 tumor B). The slopes of the plotted lines for the manual complete segmentation, manual approximation and semi-automated measurements were compared by one-way ANOVA and no significant differences among the three slopes were observed (mouse 2: P = 0.30; mouse 3 tumor A: P = 0.63; mouse 4 tumor B: P = 0.80).
(TIF)

Figure S3 **Representative micro-CT images showing a nodule that is attached to both the chest wall and diaphragm.** Micro-CT images of a nodule from mouse #3 that abuts the chest wall and diaphragm are shown. This nodule could not be successfully segmented by the semi-automated algorithm because it violates the assumption of the algorithm that the nodule can only have one major attachment. These images were windowed to enhance the contrast for viewing and representative segmentations that are seven slices apart are shown. This scan was acquired at 50 µm with 720 projections. The scale bars represent 5.0 mm. The color bar range is −625 to 1225 HU.
(TIF)

Table S1 **Scanner acquisition parameters used for live scans.**
(DOCX)

Table S2 **Comparison of tumor sizes determined by physical measurement from histological slides or by analysis of post-mortem micro-CT scans.**
(DOCX)

Table S3 **Summary of tumors detected, segmented and used from growth analysis for each mouse in live micro-CT scans.**
(DOCX)

Table S4 **Comparison of manual and semi-automated tumor volume measurements for tumors that were successfully segmented but not used in growth analysis.**
(DOCX)

Table S5 **Comparison of raw tumor volume measurements of tumors used in growth analysis by manual and semi-automated methods.**
(DOCX)

Table S6 **Descriptive statistics for the densities of the histogram plots in** **Figure 5A** **.**
(DOCX)

Table S7 **Descriptive statistics for the densities of the histogram plots in** **Figure 7** **.**
(DOCX)
