# Supplementary figures and images for: Growth Pattern Analysis of Murine Lung Neoplasms by Advanced Semi-Automated Quantification of Micro-CT Images
Source: PLoS One. 2013 Dec 23;8(12):e83806. doi: 10.1371/journal.pone.0083806 (PMC3871568; doi:10.1371/journal.pone.0083806)

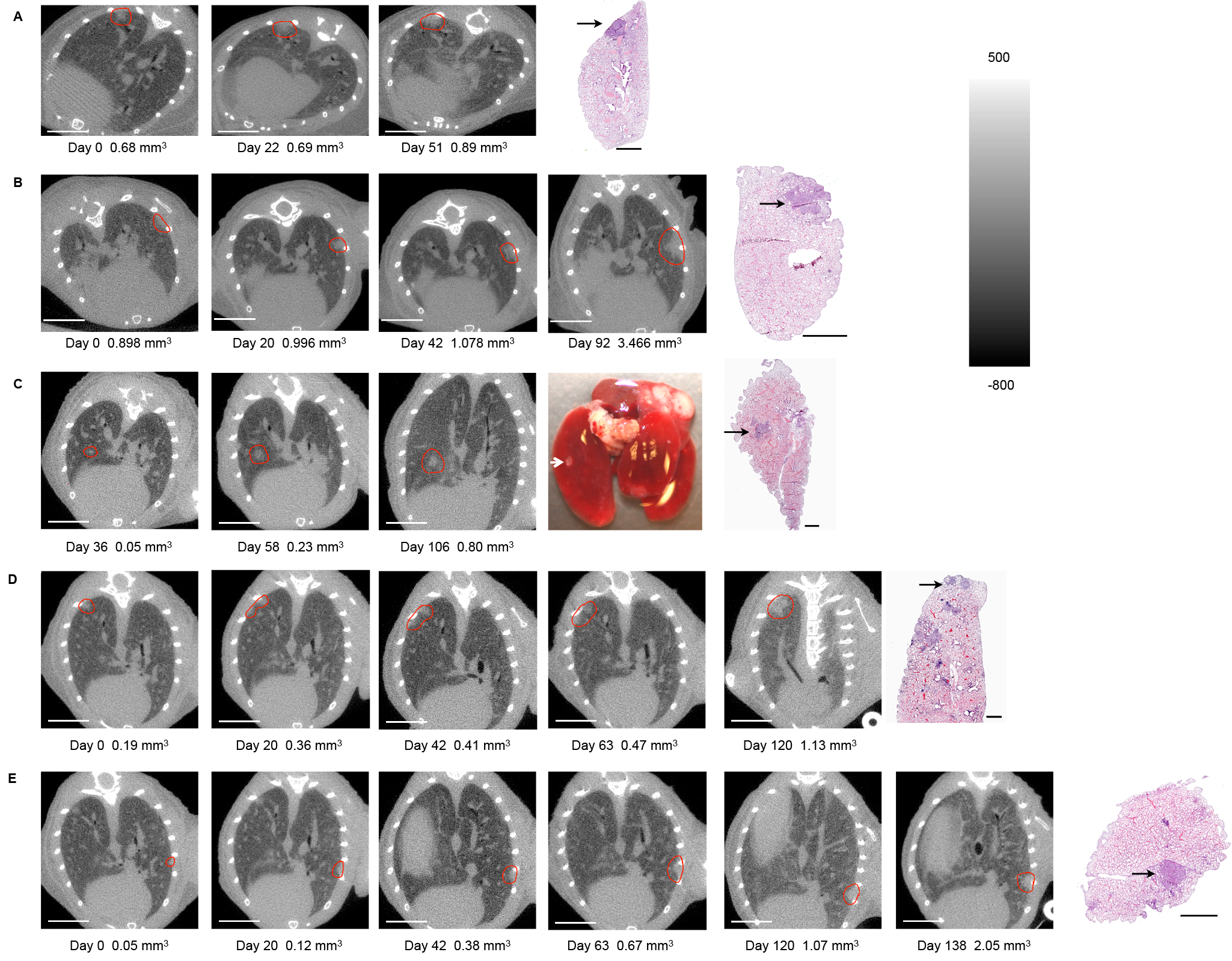

Supplement: Figure S1 — Analysis of lung tumor growth in RNR transgenic mice by sequential micro-CT scanning. Micro-CT imaging was performed on RNR transgenic mice, and representative micro-CT images from each imaging session are shown for (A) mouse #1; (B) mouse #2; (C) mouse #3 tumor B; (D) mouse #4 tumor A; (E) mouse #4 tumor B. All images were acquired at 50 µm with 720 projections. The time point of the scan and the calculated tumor volume are indicated. Manually generated red outlines highlight the tumor analyzed in each image. Also shown are H&E stained sections that were generated for each sample following the final imaging session. Scale bars represent 5.0 mm (CT images) or 1000 µm (H&E image). The color bar range is -800 to 500 HU. (TIF) [file pone.0083806.s001.tif]

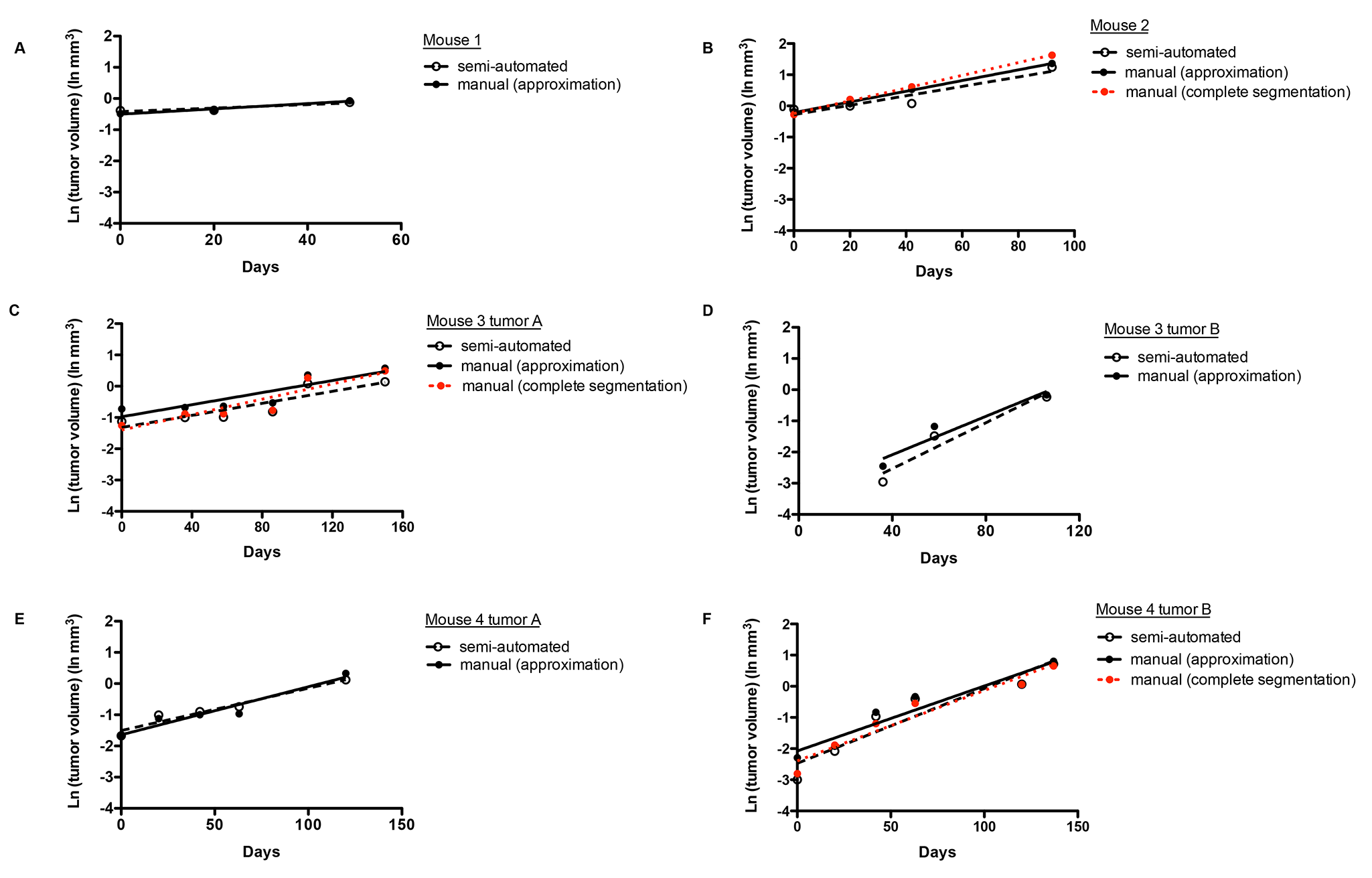

Supplement: Figure S2 — Comparison of lung tumor volume values determined manually by an observer and by the semi-automated algorithm. Four tumor-bearing mice were subjected to sequential micro-CT imaging, and tumor volume measurements of six tumors were determined manually by an observer and by the semi-automated algorithm. (A) mouse #1, (B) mouse #2, (C) mouse #3 tumor A, (D) mouse #3 tumor B, (E) mouse #4 tumor A, and (F) mouse #4 tumor B. The volumes of all six tumors were measured over time by a manual approximation method as described in Materials and Methods. The slopes of the plotted lines for the manual approximation and semi-automated measurements were compared by Student's t-test, and no significant differences between the two slopes were observed (mouse 1: P = 0.40; mouse 2: P = 0.62; mouse 3 tumor A: P = 0.99; mouse 3 tumor B: P = 0.69; mouse 4 tumor A: P = 0.57; mouse 4 tumor B: P = 0.55). In addition, the volumes of three tumors over time were further measured by manual complete 3D segmentation (mouse 2, mouse 3 tumor A and mouse 4 tumor B). The slopes of the plotted lines for the manual complete segmentation, manual approximation and semi-automated measurements were compared by one-way ANOVA and no significant differences among the three slopes were observed (mouse 2: P = 0.30; mouse 3 tumor A: P = 0.63; mouse 4 tumor B: P = 0.80). (TIF) [file pone.0083806.s002.tif]

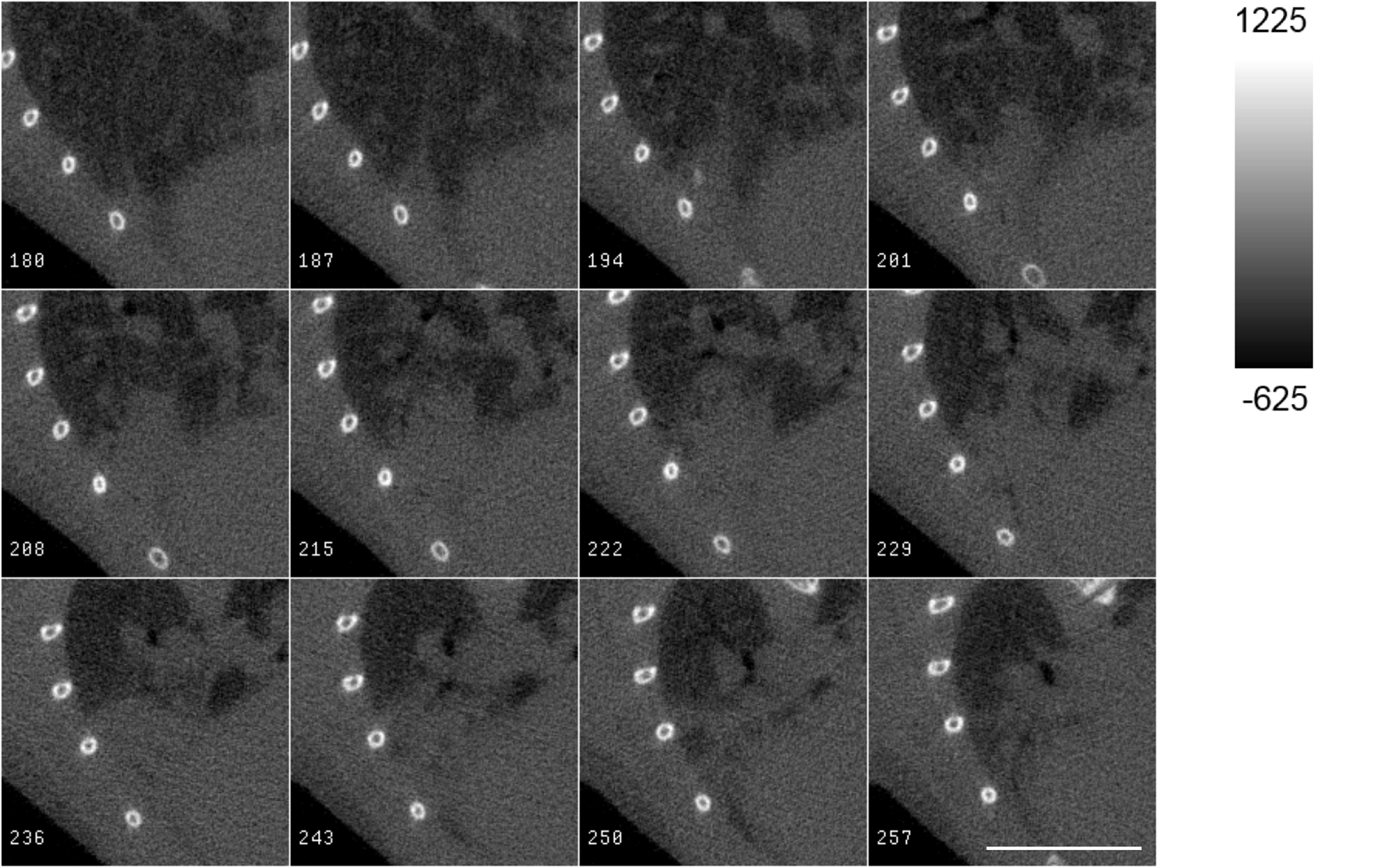

Supplement: Figure S3 — Representative micro-CT images showing a nodule that is attached to both the chest wall and diaphragm. Micro-CT images of a nodule from mouse #3 that abuts the chest wall and diaphragm are shown. This nodule could not be successfully segmented by the semi-automated algorithm because it violates the assumption of the algorithm that the nodule can only have one major attachment. These images were windowed to enhance the contrast for viewing and representative segmentations that are seven slices apart are shown. This scan was acquired at 50 µm with 720 projections. The scale bars represent 5.0 mm. The color bar range is −625 to 1225 HU. (TIF) [file pone.0083806.s003.tif]
