# Supplementary material for: Growth Pattern Analysis of Murine Lung Neoplasms by Advanced Semi-Automated Quantification of Micro-CT Images
Source: PLoS One. 2013 Dec 23;8(12):e83806. doi: 10.1371/journal.pone.0083806 (PMC3871568; doi:10.1371/journal.pone.0083806)
Supplement: Table S1 — Scanner acquisition parameters used for live scans. (DOCX) [file pone.0083806.s004.docx]

**Table S1^a^. Scanner acquisition parameters used for live scans.**

|  | **Current (mA)** | **Voltage (kVp)** | **Exposure Time (ms)** | **# of Views/scan** | **Frame averaging** |
| --- | --- | --- | --- | --- | --- |
| All except | 50 | 100 | 20 | 720 | 2 |
| Mouse 1 (time point 3) | 50 | 100 | 20 | 720 | 1 |
| Mouse 2 (time point 1) | 50 | 100 | 20 | 440 | 2 |

**^a^** These live scans were acquired and reconstructed at 50 µm resolution.
