# Supplementary material for: Growth Pattern Analysis of Murine Lung Neoplasms by Advanced Semi-Automated Quantification of Micro-CT Images
Source: PLoS One. 2013 Dec 23;8(12):e83806. doi: 10.1371/journal.pone.0083806 (PMC3871568; doi:10.1371/journal.pone.0083806)
Supplement: Table S2 — Comparison of tumor sizes determined by physical measurement from histological slides or by analysis of post-mortem micro-CT scans. (DOCX) [file pone.0083806.s005.docx]

**Table S2. Comparison of tumor sizes determined by physical measurement from histological slides or by analysis of post-mortem micro-CT scans.**

| **Mouse (tumor)** | **Diameter by physical measurement of H&E section^a^(mm)** | **Diameter by manual measurement of CT scan^b^ (mm)** | **Diameter by semi-automated measurement of CT scan^c^ (mm)** | **Percent difference between manual and semi-automated methods^d^** |
| --- | --- | --- | --- | --- |
| Mouse 1 (tumor A) | 1.94 | 2.06 | 2.03 | 1.47% |
| Mouse 1 (tumor B) | 1.89 | 1.74 | 1.82 | 4.49% |
| Mouse 2 | 0.72 | 1.24 | 1.28 | 3.17% |
| Mouse 3 | 2.39 | 1.76 | 1.73 | 1.72% |

**^a^** Physical measurements of tumor diameters were made from H&E-stained histological sections (see Materials and Methods for detailed measurement methods). Values are for the largest diameter of the tumor.

**^b^** Tumor volumes were measured manually from micro-CT scans (see Materials and Methods for detailed manual approximation measurement methods). For ease of comparison, the volume measured manually was converted to an equivalent diameter assuming the tumor is a sphere.

**^c^** The semi-automated algorithm is not designed for diameter measurement. Rather, for ease of comparison, the volume measured by the algorithm was converted to an equivalent diameter assuming the tumor is a sphere.

**^d^** The percent difference between the tumor diameter measurements made with the manual and semi-automated methods was calculated as a way to compare the two methods. The difference between the two diameters was divided by the average of the two diameters, and then converted to a percentage.
