# Supplementary material for: Growth Pattern Analysis of Murine Lung Neoplasms by Advanced Semi-Automated Quantification of Micro-CT Images
Source: PLoS One. 2013 Dec 23;8(12):e83806. doi: 10.1371/journal.pone.0083806 (PMC3871568; doi:10.1371/journal.pone.0083806)
Supplement: Table S3 — Summary of tumors detected, segmented and used from growth analysis for each mouse in live micro-CT scans. (DOCX) [file pone.0083806.s006.docx]

**Table S3. Summary of tumors detected, segmented and used for growth analysis for each mouse in live micro-CT scans**

| **Mouse Number** | **Total tumors detected** | **Total tumors segmented** | **Total tumors used for growth analysis** |
| --- | --- | --- | --- |
| Mouse 1 | 3 | 3 | 1 |
| Mouse 2 | 2 | 2 | 1 |
| Mouse 3 | 4 | 3 | 2 |
| Mouse 4 | 3 | 2 | 2 |
