# Supplementary material for: Growth Pattern Analysis of Murine Lung Neoplasms by Advanced Semi-Automated Quantification of Micro-CT Images
Source: PLoS One. 2013 Dec 23;8(12):e83806. doi: 10.1371/journal.pone.0083806 (PMC3871568; doi:10.1371/journal.pone.0083806)
Supplement: Table S4 — Comparison of manual and semi-automated tumor volume measurements for tumors that were successfully segmented but not used in growth analysis. (DOCX) [file pone.0083806.s007.docx]

**Table S4^a^. Comparison of manual and semi-automated tumor volume measurements for tumors that were successfully segmented but not used in growth analysis.**

| **Tumor Number** | **Manual measurement (mm^3^)** | **Semi-automated measurement (mm^3^)** |
| --- | --- | --- |
| Mouse 1 tumor B | 1.058 | 1.129 |
| Mouse 1 tumor C | 0.578 | 0.624 |
| Mouse 2 tumor B | 0.152 | 0.114 |
| Mouse 3 tumor C time point 1 | 0.128 | 0.132 |
| Mouse 3 tumor C time point 2 | 0.178 | 0.158 |

^a^ The tumor volumes measured by the manual approximation and semi-automated methods were compared and tested by paired Student’s t-test and no significant difference was observed (P=0.57).
