# Supplementary material for: Growth Pattern Analysis of Murine Lung Neoplasms by Advanced Semi-Automated Quantification of Micro-CT Images
Source: PLoS One. 2013 Dec 23;8(12):e83806. doi: 10.1371/journal.pone.0083806 (PMC3871568; doi:10.1371/journal.pone.0083806)
Supplement: Table S5 — Comparison of raw tumor volume measurements of tumors used in growth analysis by manual and semi-automated methods. (DOCX) [file pone.0083806.s008.docx]

**Table S5^a^. Comparison of raw tumor volume measurements of tumors used in growth analysis by manual and semi-automated methods.**

| Mouse (tumor) | Tumor volume from semi-automated method (mm^3^) | Tumor volume from semi-automated method (mm^3^) | Tumor volume from manual approximation method (mm^3^) | Tumor volume from manual complete segmentation method (mm^3^) |
| --- | --- | --- | --- | --- |
| 1 | 0 | 0.685 | 0.615 |  |
|  | 20 | 0.688 | 0.700 |  |
|  | 49 | 0.891 | 0.928 |  |
| 2 | 0 | 0.898 | 0.833 | 0.759 |
|  | 20 | 0.996 | 1.070 | 1.229 |
|  | 42 | 1.078 | 1.704 | 1.843 |
|  | 92 | 3.466 | 3.905 | 5.079 |
| 3 (A) | 0 | 0.324 | 0.485 | 0.285 |
|  | 36 | 0.368 | 0.506 | 0.413 |
|  | 58 | 0.372 | 0.527 | 0.415 |
|  | 86 | 0.442 | 0.589 | 0.462 |
|  | 106 | 1.087 | 1.425 | 1.306 |
|  | 150 | 1.151 | 1.776 | 1.635 |
| (B) | 36 | 0.052 | 0.086 |  |
|  | 58 | 0.229 | 0.308 |  |
|  | 106 | 0.800 | 0.847 |  |
| 4 (A) | 0 | 0.188 | 0.187 |  |
|  | 20 | 0.364 | 0.326 |  |
|  | 42 | 0.408 | 0.370 |  |
|  | 63 | 0.476 | 0.379 |  |
|  | 120 | 1.130 | 1.390 |  |
| (B) | 0 | 0.050 | 0.100 | 0.061 |
|  | 20 | 0.120 | 0.146 | 0.152 |
|  | 42 | 0.380 | 0.434 | 0.302 |
|  | 63 | 0.670 | 0.716 | 0.578 |
|  | 120 | 1.070 | 1.099 | 1.061 |
|  | 137 | 2.050 | 2.230 | 1.920 |

^a^ For tumors that had all three volume measurements (16 in total), a one way ANOVA test was performed and no significant difference was detected among the three datasets (P=0.06); for tumors that had only the semi-automated and manual approximation measurements (11 in total), a paired Student’s t-test was performed and no significant difference was observed between the two datasets (P=0.49).
