# Supplementary material for: Growth Pattern Analysis of Murine Lung Neoplasms by Advanced Semi-Automated Quantification of Micro-CT Images
Source: PLoS One. 2013 Dec 23;8(12):e83806. doi: 10.1371/journal.pone.0083806 (PMC3871568; doi:10.1371/journal.pone.0083806)
Supplement: Table S6 — Descriptive statistics for the densities of the histogram plots in Figure 5A . (DOCX) [file pone.0083806.s009.docx]

**Table S6^a^. Descriptive statistics for the densities of the histogram plots in Figure 5A.**

|  | **Air** | | **Water** | | **Bone** | |
| --- | --- | --- | --- | --- | --- | --- |
| **Scan** | **Mean (HU)** | **SD (HU)** | **Mean (HU)** | **SD (HU)** | **Mean (HU)** | **SD (HU)** |
| Mouse 4 (Time point 2) | -927.6 | 77.8 | 92.2 | 82.3 | 2612.6 | 120.6 |
| Mouse 4 (Time point 4) | -931.8 | 108.7 | 66.5 | 107.0 | 2592.0 | 124.0 |

**^a^** The densities of ten 350 μm x 350 μm x 150 μm regions in each material were sampled and used to compute the standard deviation (SD). SD is a measure of the noise in the scan, with higher values indicating more noise.
