# Supplementary material for: Growth Pattern Analysis of Murine Lung Neoplasms by Advanced Semi-Automated Quantification of Micro-CT Images
Source: PLoS One. 2013 Dec 23;8(12):e83806. doi: 10.1371/journal.pone.0083806 (PMC3871568; doi:10.1371/journal.pone.0083806)
Supplement: Table S7 — Descriptive statistics for the densities of the histogram plots in Figure 7 . (DOCX) [file pone.0083806.s010.docx]

**Table S7^a^. Descriptive statistics for the densities of the histogram plots in Figure 7.**

|  | Parenchyma | | Soft tissue | |
| --- | --- | --- | --- | --- |
|  | Mean | SD | Mean | SD |
| **Micro-CT** | -376.9 | 110.2 | -1.6 | 109.4 |
| **Human CT** | -883.8 | 79.0 | 57.9 | 82.3 |

**^a^** The densities of small regions within the lung parenchyma were sampled and used to compute the standard deviation (SD). SD is a measure of the noise in the scan, with higher values indicating more noise.
